# IFNAR2 Is Required for Anti-influenza Immunity and Alters Susceptibility to Post-influenza Bacterial Superinfections

**DOI:** 10.3389/fimmu.2018.02589

**Published:** 2018-11-09

**Authors:** Kelly M. Shepardson, Kyle Larson, Laura L. Johns, Kayla Stanek, Hanbyul Cho, Julia Wellham, Haley Henderson, Agnieszka Rynda-Apple

**Affiliations:** Rynda-Apple Laboratory, Department of Microbiology and Immunology, Montana State University, Bozeman, MT, United States

**Keywords:** IFNAR2, IFNAR1, Influenza, *Staphylococcus aureus*, superinfection

## Abstract

Influenza virus infections particularly when followed by bacterial superinfections (BSI) result in significant morbidities and mortalities especially during influenza pandemics. Type I interferons (IFNs) regulate both anti-influenza immunity and host susceptibility to subsequent BSIs. These type I IFNs consisting of, among others, 14 IFN-α's and a single IFN-β, are recognized by and signal through the heterodimeric type I IFN receptor (IFNAR) comprised of IFNAR1 and IFNAR2. However, the individual receptor subunits can bind IFN-β or IFN-α's independently of each other and induce distinct signaling. The role of type I IFN signaling in regulating host susceptibility to both viral infections and BSI has been only examined with respect to IFNAR1 deficiency. Here, we demonstrate that despite some redundancies, IFNAR1 and IFNAR2 have distinct roles in regulating both anti-influenza A virus (IAV) immunity and in shaping host susceptibility to subsequent BSI caused by *S. aureus*. We found IFNAR2 to be critical for anti-viral immunity. In contrast to *Ifnar1*^−/−^ mice, IAV-infected *Ifnar2*^−/−^ mice displayed both increased and accelerated morbidity and mortality compared to WT mice. Furthermore, unlike IFNAR1, IFNAR2 was sufficient to generate protection from lethal IAV infection when stimulated with IFN-β. With regards to BSI, unlike what we found previously in *Ifnar1*^−/−^ mice, *Ifnar2*^−/−^ mice were not susceptible to BSI induced on day 3 post-IAV, even though absence of IFNAR2 resulted in increased viral burden and an increased inflammatory environment. The *Ifnar2*^−/−^ mice similar to what we previously found in *Ifnar1*^−/−^ mice were less susceptible than WT mice to BSI induced on day 7 post-IAV, indicating that signaling through a complete receptor increases BSI susceptibility late during clinical IAV infection. Thus, our results support a role for IFNAR2 in induction of anti-IAV immune responses that are involved in altering host susceptibility to BSI and are essential for decreasing the morbidity and mortality associated with IAV infection. These results begin to elucidate some of the mechanisms involved in how the individual IFNAR subunits shape the anti-viral immune response. Moreover, our results highlight the importance of examining the contributions of entire receptors, as individual subunits can induce distinct outcomes as shown here.

## Introduction

Influenza A virus (IAV) causes one of the most common respiratory infections worldwide. While infection from IAV can be detrimental on its own, more typically, anti-viral immune responses induced during IAV infection alter host susceptibility to bacterial superinfections (BSI). Over the past decades, *Staphylococcus aureus* has become one of the predominant bacteria involved in BSIs leading to increased morbidity and mortality, especially during IAV pandemics (1–3). A better understanding of the anti-viral immune factors that are involved in altering the host susceptibility to BSI is essential for decreasing the morbidity and mortality associated with post-IAV BSI.

It is well-known that type I IFNs, including a single IFN-β and 14 IFN-α's, have an established role in the anti-viral immunity to influenza and are known to regulate host susceptibility to the subsequent BSIs. Because type I IFNs are thought to require recognition by the heterodimeric IFNAR1/IFNAR2 receptor, depletion of the IFNAR1 subunit (*Ifnar1*^−/−^) is assumed to render mice unresponsive to type I IFN signaling. However, previous studies on the role of IFNAR1 in influenza infection and post-influenza BSI models showed conflicting results. Absence of IFNAR1 either in knockout mice or by antibody blockage resulted in a range of responses. These responses varied from increased mouse susceptibility to infection (4–7), no significant antiviral effect (8), no effect on survival from influenza disease (9), or reduced susceptibility by the means of better controlled inflammation (10). Recent reports have also demonstrated that IFN-β can ligate to either IFNAR1 or IFNAR2 individually, while IFN-α appeared to only ligate to IFNAR2, and that the two different IFN-β/IFNAR subunit complexes can transduce expression of distinct sets of genes (11). Additional evidence for the existence of differential signaling by IFNAR1 and IFNAR2 is supported by an absence of sepsis morbidity in *Ifnar1*^−/−^ mice compared to high morbidity in *Ifnar2*^−/−^ and WT mice. These conflicting results prompted us to investigate whether the individual IFNAR subunits are sufficient for interferon signaling, and whether they have non-redundant roles in mediating host immunity to IAV infections and subsequent BSIs. Here, we report that despite some redundancies, IFNAR1 and IFNAR2 have distinct roles in regulating both anti-IAV immunity and in shaping host susceptibility to a subsequent *S. aureus* BSI. Importantly, these results highlight the need to understand the contribution of individual IFNAR subunits to the infections mediated by either virus or bacteria alone, or together in the context of BSI.

## Materials and methods

### Mice

Male and female wild type (WT) C57BL/6 (CD45.2), *Ifnar1*^−/−^, and *Ifnar2*^−/−^ (Ifnar2tm1(KOMP)Vlcg)mice (originally purchased from Jackson Laboratories or UC Davis KOMP Repository) were bred and maintained at Montana State University (Bozeman, MT) Animal Resources Center under pathogen-free conditions. All mice used in this study were between 6 and 8 weeks of age at the initiation of experiment. Mice were weighed and monitored for signs of morbidity and mortality. All care and procedures were in accordance with the recommendations of the NIH, the USDA, and the *Guide for the Care and Use of Laboratory Animals* (8th ed.) (12). Animal protocols were reviewed and approved by the MSU Institutional Animal Care and Use Committee (IACUC). MSU is accredited by the Association for Assessment and Accreditation of Laboratory Animal Care (AAALAC; number 713).

### Inoculations and challenge

Nonsurgical intratracheal (i.t.) inoculations were performed as described previously for all inoculations and challenges (13). Virus inoculation: mice were inoculated with 100 μL of PBS or 0.1LD_50_ (1,500 plaque forming units [PFU]) of IAV strain A/PR8/8/34 (PR8; H1N1). Bacterial challenge: mice were inoculated with 100 μL of PBS or 10^8^ CFU of LAC strain of *S. aureus* (MRSA pulsed-field type USA300; was a kind gift from Jovanka Voyich at MSU). Our previously described procedure for determining CFUs (13) and PFUs (14) was followed on lung homogenate samples. Phosphorylation of STAT3 (P-STAT3) inhibition: mice were inoculated 6 h after IAV infection with 100 μL containing 100 μg of FLLL32 [Cayman Chemical, (15)] in 10% DMSO. Control mice were inoculated with equal volume of PBS with 10% DMSO. Mouse recombinant IFN inoculation (mrIFN): mice were inoculated with either 10^4^ IU mrIFN-β (PBLassay bioscience) or with 2.5 × 10^5^ IU mrIFN-αA in 100 μL PBS (BioLegend) at 0, 3, and 6 h post-IAV infection.

### Preparation of balf samples and cytokine analyses

Mice were sacrificed by intraperitoneal (i.p.) administration of 90 mg/kg of body weight sodium pentobarbital. Bronchoalveolar lavage (BAL) was performed by washing the lungs with 3 mM EDTA in PBS (16) and cellular composition was determined by hemocytometer cell counts and differential counts of cytospins after staining with Quick-Diff solution (Siemens; Medical Solutions Diagnostics, Tarrytown, NY). Cell-free BAL fluid (BALF) was used to determine levels of IL-13 (4–500 pg/ml) and IFN-α (31.3–20.00 pg/ml) using ELISA kits (Ready-SET-Go; eBioscience, San Diego, CA), and levels of IL-1α (Minimal Detectable Concentration (MDC); 1.3 pg/mL), IFNγ (MDC; 0.8 pg/mL), TNFα (MDC; 1.9 pg/mL), IL-1β (MDC; 2.8 pg/mL), IL-10 (MDC; 2.1 pg/mL), IL-6 (MDC; 0.9 pg/mL), IFN-β (MDC; 4 pg/mL) using the LEGENDplex mouse inflammation panel (BioLegend, San Diego, CA). LEGENDplex panel was acquired on LSRII running FACS-Diva software (both obtained from BD Bioscience) and analyzed using BioLegend data analysis software. Results are from ≥4 mice per group (biological replicates) and 2 technical replicates per mouse.

### qRT-PCR

Mice were inoculated with IAV or PBS as described above and euthanized 24 h after inoculation. Lungs were homogenized and RNA was extracted immediately using Trizol reagent and chloroform method per manufacturer's protocol. RNA was reverse transcribed with QuantiTect reverse transcription kit (Qiagen, USA). Primers for all murine genes of interest were designed, unless denoted, with PrimerQuest (IDT) and all were manufactured by IDT, USA. Sequences are:

STAT3 (17) Fwd: GGATCGCTGAGGTACAACCC
  STAT3 Rev: GTCAGGGGTCTCGACTGTCT
  STAT6 (18) Fwd: TCTCCACGAGCTTCACATTG
  STAT6 Rev: GACCACCAAGGGCAGAGAC
  STAT1 Fwd: GACCCTAAGCGAACTGGATAC
  STAT1 Rev: TGTCGCCAGAGAGAAATTCGTGT
  STAT2 Fwd: CGGCCAACAGGTGAAATTAAG
  STAT2 Rev: GGGACTTACAAAGGAGCAGAA
  IRF3 Fwd: CCCACAGTGCTACTGATACC
  IRF3 Rev: GTCACACCAGACTTAGGAATGT
  IRF7 Fwd: TATGCAAGGCATACCTGGAG
  IRF7 Rev: CGATGTCTTCGTAGAGACTGTT

rpl13a fwd: CTCTGGAGGAGAAACGGAAGGAAA, rev: GGTCTTGAGGACCTCTGTGAACTT. All reactions were performed on Roche LightCycler 96 real-time PCR detection system with iTaq universal SYBR green supermix (Bio-Rad, Hercules, CA). The ΔΔC_t_ method was used to assess changes in mRNA abundance, using rpl13a as the housekeeping gene. Results presented are the mean and standard deviation from three biological and three experimental replicates.

### Survival, morbidity, LDH, and albumin

Mice were weighed on a daily basis and assessed for signs of morbidity and mortality. Morbidity measures were as follow: 0, normal; 1, hunched back or ruffled fur; 2, both hunched back and ruffled fur; 3, not moving over a 5 min period. Lactate dehydrogenase (LDH) in the cell-free BALF was measured using the CytoTox 96^®;^ Non-Radioactive Cytotoxicity Assay (Promega) and albumin in the cell-free BALF was measured using QuantiChrom BCG Albumin Assay Kit (BioAssay Systems) following the manufacturers protocols.

### Primary alveolar epithelial cell harvest

After mice were euthanized, their lungs were perfused with PBS containing gentamycin (10 μg/mL) injected into the heart. Lungs were then lavaged with PBS containing 3 mM EDTA, filled with 1 mL PBS containing Dispase (50 units/mL) and Elastase (5 units/mL) to degrade extracellular matrix proteins and tied off. Lungs were incubated at 37°C while shaking for 1 h and following incubation elastase activity was terminated by addition of fetal bovine serum (FBS) and Dnase (4 units/mL) was also added to the lung homogenate samples to ensure degradation of extracellular DNA. The lungs were then dissected and aspirated through syringes to create a single cell suspension. The cell suspension was filtered (100 and 50 μm) and separated by a discontinuous Percoll gradient consisting of a heavy layer (5.2 mL Percoll, 50 μL FBS, 3.8 mL water, 1 mL 10 × PBS/100 mM HEPES/55 mM Glucose/pH 7.4) and a light layer (3.6 mL Percoll, 50 μL FBS, 6 mL HBSS, 0.4 10 × PBS/100 mM HEPES/55 mM Glucose/pH 7.4) by centrifuging at 2,000 rpm for 20 min. Cells were collected, washed and non-epithelial cells were removed by incubating collected cells in a plate pre-coated with IgG (5 ug/mL) for 30 min. Epithelial cells were collected, washed and transferred to plates pre-coated with fibronectin (Calbiochem, 50 μg/mL) in DMEM/F-12 media (Hyclone) containing 1.5 g/L NAHCO_3_, 5 mL insulin/transferrin/sodium selenite (Gibco), 1 μg/mL hydrocortisone (Sigma), 1 mM L-glutamine, 10% FBS, and Pen-Strep. Upon confluency, cells were passaged and expanded for use.

### Western blot

For whole lung protein fractions, mice were euthanized 24 h after inoculation as described above, lungs were homogenized, and protein was extracted using RIPA buffer (50 mM Tris-HCl pH 7.5, 2 mM EDTA, 150 mM NaCl, 1% NP-40, 0.1% SDS, 0.5% Na-deoxycholate, 50 mM NaF). Primary alveolar epithelial cells were grown to ~80–90% confluence on T-75 flasks and were treated with IAV (MOI 1) or PBS. At 24 h after treatment cells were washed with ice-cold PBS and cytoplasmic protein fraction was extracted with Buffer A (10 mM HEPES pH 8.0, 0.5% NP-40, 1.5 mM MgCl_2_, 10 mM KCl, 0.5 mM DTT, and 200 mM sucrose). The tubes were incubated for 30 min on ice, and then centrifuged at 14,000 rpm at 4°C and the supernatant was collected. The pellets were resuspended in 30 μl of RIPA buffer and incubated on ice for 30 min. Then they were centrifuged at 14,000 rpm at 4°C and the supernatant was collected as the nuclear protein fraction. Proteins were quantified using BCA (Pierce). Proteins were run on a 10% SDS-Page gel, transferred to nitrocellulose, and stained for either STAT3 (Cell Signaling; D1B2), P-STAT3 (Cell Signaling; Tyr705, D3A7), or beta-actin (BioLegend; Poly6221). Protein levels were normalized to beta-actin and fold change was calculated over WT PBS.

### Statistical analyses

Reported results are means ± SD of ≥4 mice/group from a single experiment. Each experiment for which results are presented in the manuscript was independently performed at least twice with similar results. The differences between treatment groups were analyzed by analysis of variance (ANOVA) or Student's *t-*test (two-tailed) using GraphPad Prism software V.7.0d. For the differences in survival Kaplan-Meier curves were plotted and analyzed using GraphPad Prism software (Version 4.0; La Jolla, CA) using Gehan-Breslow-Wilcoxon (Logrank) test. Statistical differences with *P* < 0.05 were considered significant.

## Results

### Absence of IFNAR2 causes increased morbidity and mortality in response to IAV infection

**Figure 1:**
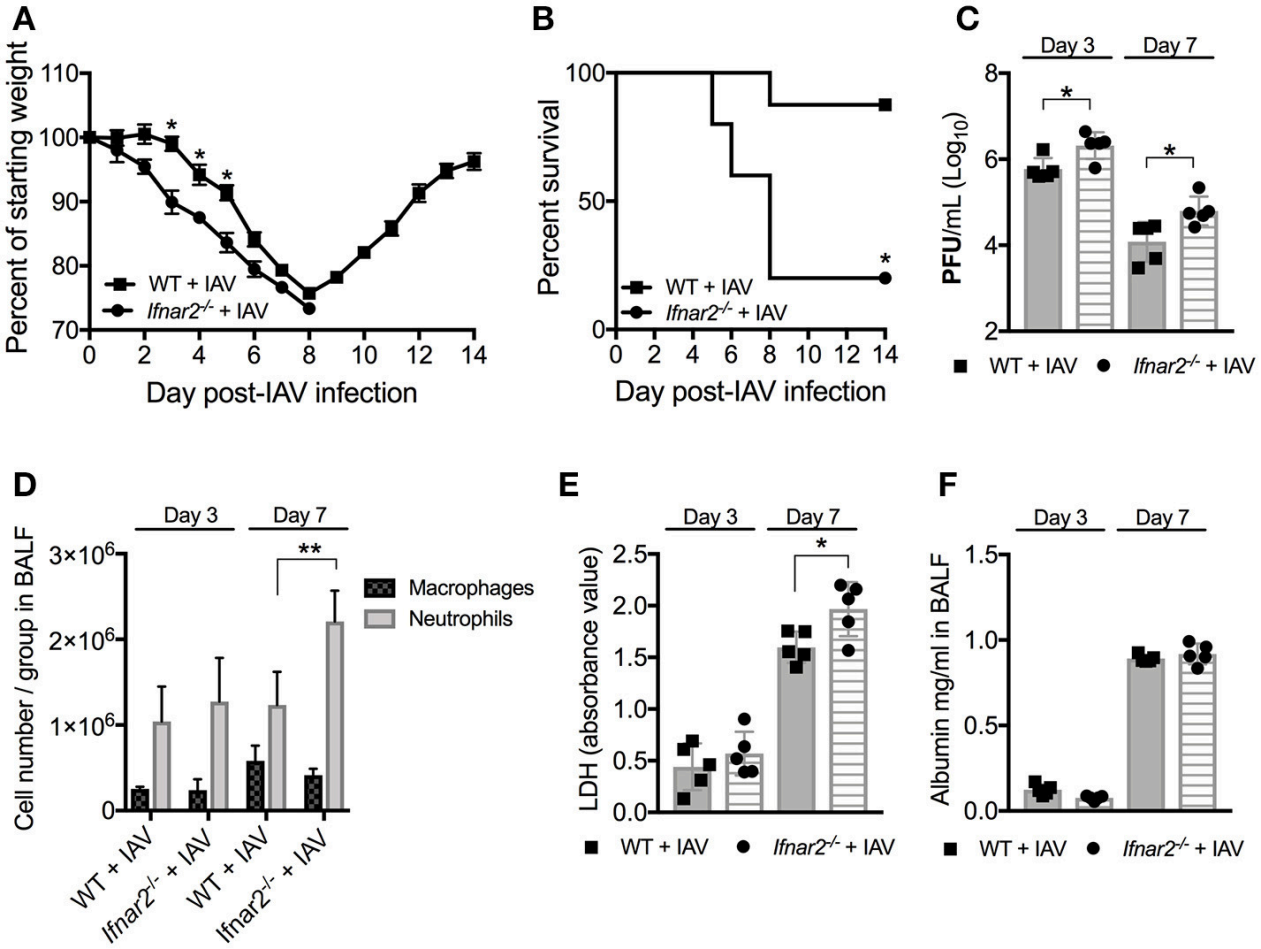
*Ifnar2*^−/−^ mice are more susceptible to IAV. WT and *Ifnar2*^−/−^ mice were infected with IAV or PBS on day 0. **(A)** Weights were monitored daily and are represented as percent of starting body weight depicted as average/group (Significance represents WT+IAV compared to *Ifnar2*^−/−^+IAV). **(B)** Survival is represented as percent survival/group. PFUs **(C)** were determined on day 3 or day 7 post-IAV. **(D)** Differential counts were determined from cell-free BALF on day 3 or day 7 post-IAV. LDH **(E)** and Albumin **(F)** were analyzed from cell-free BALF on day 3 or day 7 post-IAV. Data shown are mean ± SD results of ≥4 animals per group from one representative experiment. **P >0.01; *P > 0.05.

It is well-known that type I IFNs are involved in anti-viral immunity to influenza and involved in BSI outcome. As type I IFNs are recognized by and signal through the heterodimeric IFNAR1/IFNAR2 receptor, depletion of the IFNAR1 subunit (*Ifnar1*^−/−^) has been assumed to eliminate type I IFN signaling in mice. However, IFN-β has recently been shown to ligate the IFNAR subunits independently resulting in distinct gene induction (11), suggesting that the individual receptors may have distinct functions following IFN recognition. Since the *Ifnar2*^−/−^ mice have not been characterized in terms of susceptibility to IAV or BSI, we first sought to determine the role of IFNAR2 throughout the course of IAV infection. We found that *Ifnar2*^−/−^ mice were more susceptible to IAV infection than WT mice indicated by earlier weight loss (Figure 1A) and significantly reduced survival (Figure 1B). Unlike *Ifnar1*^−/−^ mice, that in our hands had similar viral load to WT mice throughout influenza disease (9), *Ifnar2*^−/−^ mice had increased viral burden on both days 3 and 7 of IAV infection compared to WT mice (Figure 1C). These and our previously published results imply that the presence of IFNAR2 is sufficient for controlling viral infection in *Ifnar1*^−/−^ mice (9), but the presence of IFNAR1 in *Ifnar2*^−/−^ mice is not. Both weight loss and morbidity can be due to increased cellular recruitment into the lung, but also inflammation resulting from cellular and/or tissue damage. At day 3 post-IAV, when the virus titer is typically at its peak (19), neither the pattern of cellular recruitment into the lung (Figure 1D) nor the extent of lung damage as measured by LDH (Figure 1E) and albumin (Figure 1F) were different between *Ifnar2*^−/−^ and WT mice. This suggests that IFNAR2 deficiency does not affect cell recruitment or lung integrity early during IAV infection. At day 7 post-IAV, when mice experience the peak of body weight loss, *Ifnar2*^−/−^ mice had increased overall cell numbers with significantly more neutrophils recruited to the lung compared to WT mice (Figure 1D). The increased number of neutrophils in *Ifnar2*^−/−^ mice corresponded to an increase in LDH (Figure 1E), but not in albumin (Figure 1F) when compared to WT mice.

### Absence of IFNAR2 alters the inflammatory environment during IAV

**Figure 2:**
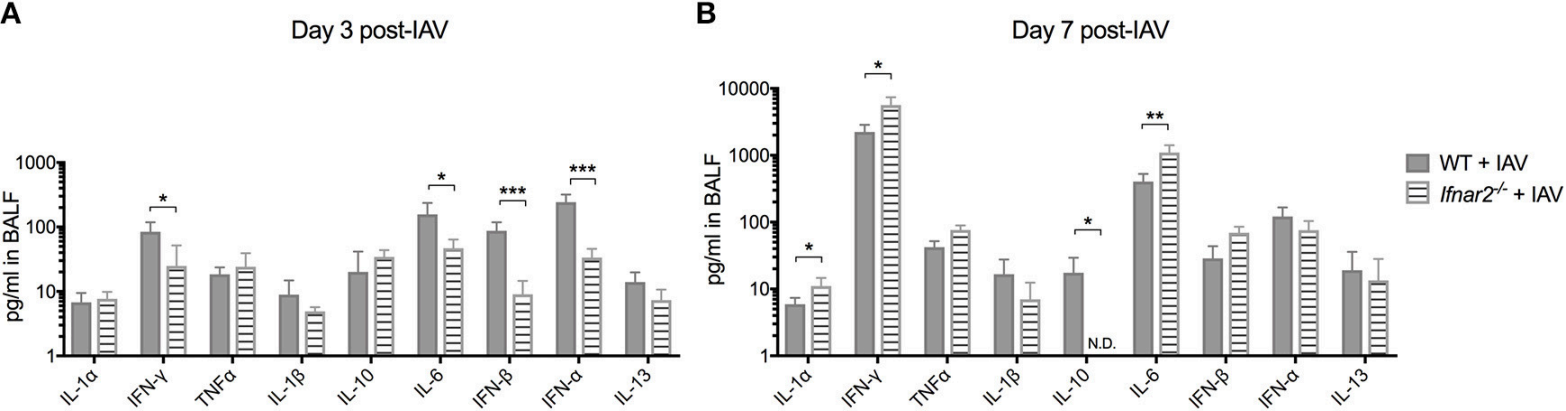
Absence of IFNAR2 initially reduces the inflammatory environment during IAV. WT and *Ifnar2*^−/−^ mice were infected with IAV or PBS on day 0. Indicated cytokines were measured in cell-free BALF collected on **(A)** day 3 or **(B)** day 7 post-IAV. Data shown are mean ± SD results of ≥4 animals per group from one representative experiment. N.D. is not detectable. **P >0.01; *P > 0.05.

Due to the impaired anti-viral immune response in *Ifnar2*^−/−^ mice and enhanced neutrophil recruitment later during IAV infection, we sought to determine whether these mice exhibit reduced anti-viral cytokines in the lung throughout IAV infection. At day 3 post-IAV we found a decrease in IFNγ and IL-6 levels in the *Ifnar2*^−/−^ mice compared to WT mice (Figure 2A). The *Ifnar2*^−/−^ mice at day 3 post-IAV also had about 1 log less of each IFN-β and IFN-α in their lungs compared to WT mice. At day 7 post-IAV there was an increase in the inflammatory cytokines IL-1α, IFNγ, and IL-6 and a decrease in the anti-inflammatory cytokine IL-10 compared to WT mice (Figure 2B). The increase in inflammatory cytokines at day 7 post-IAV in *Ifnar2*^−/−^ mice compared to WT mice correlated with the increased neutrophil recruitment and cellular damage (LDH) found in these mice at that time (Figures 1D,E). These results imply that induction of a proinflammatory response early after IAV infection is reduced in the absence of IFNAR2, whereas later after IAV, lack of IFNAR2 resulted in exacerbated lung inflammation.

### Absence of IFNAR2 signaling limits IAV burden, but does not alter susceptibility to BSI at day 3 Post-IAV

**Figure 3:**
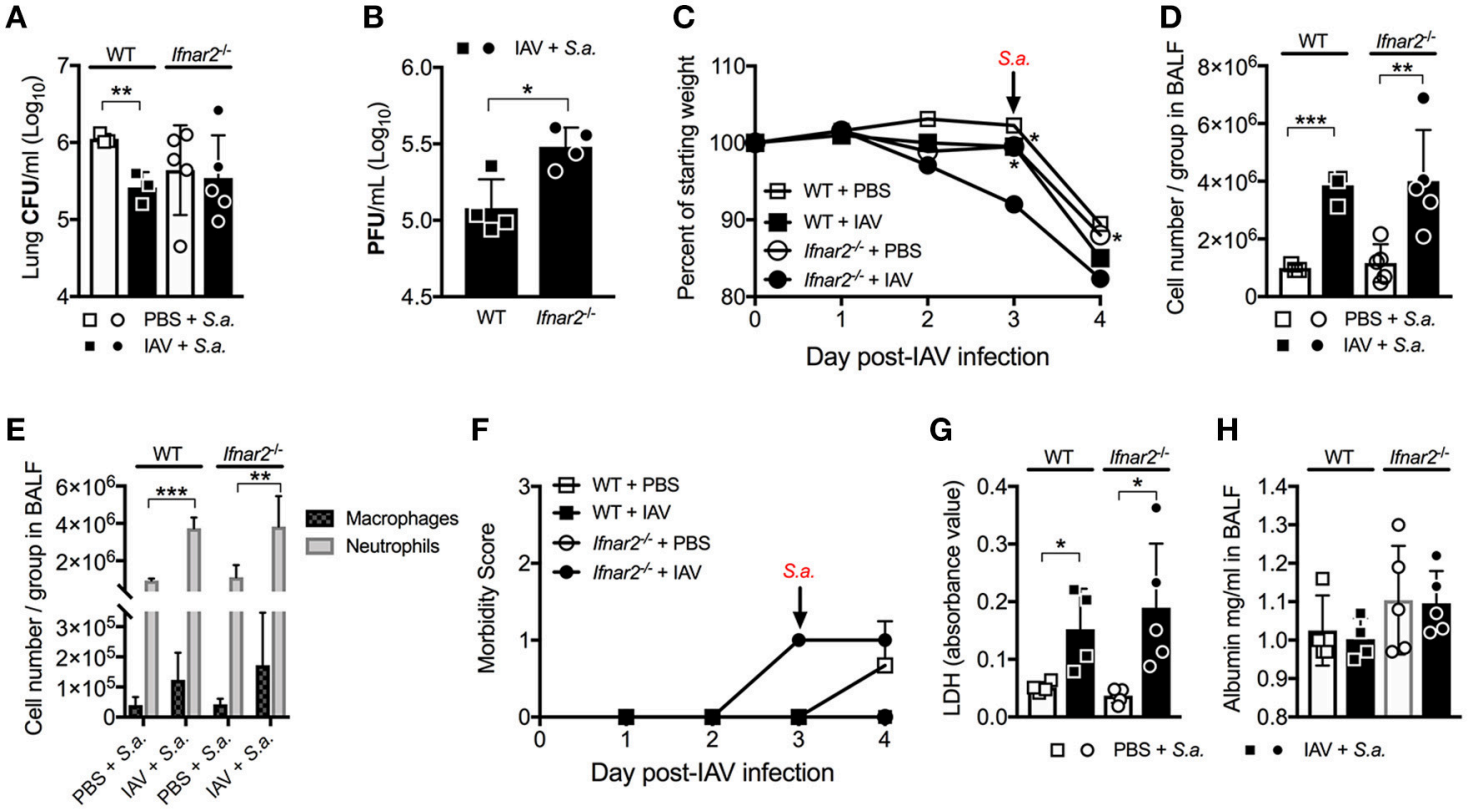
Absence of IFNAR2 does not alter susceptibility to day 3 BSI post-IAV. WT and *Ifnar2*^−/−^ mice were infected with IAV or PBS on day 0 and challenged with *S. aureus* on day 3. Lung CFUs **(A)** and PFUs **(B)** were determined 24 h post-*S.a*.-challenge. **(C)** Weights were monitored daily and are represented as percent of initial body weight depicted as average/group (Significance represents WT+IAV or *Ifnar2*^−/−^ compared to *Ifnar2*^−/−^+IAV). Cell number **(D)** and differential counts **(E)** were determined from cell-free BALF 24 h post- *S.a*.-challenge. **(F)** Mice were monitored and scored daily for signs of morbidity as described in methods section. Average daily score for each group is depicted. LDH **(G)** and Albumin **(H)** were analyzed in the cell-free BALF collected at 24 h post- *S.a*.-challenge. Data shown are mean ± SD results of ≥4 animals per group from one representative experiment. ***P >0.001; **P >0.01; *P > 0.05.

Previously we found that while IFNAR1 was not required for anti-IAV immunity (9), it was important for controlling host susceptibility to *S. aureus* BSI. Specifically, we demonstrated that *Ifnar1*^−/−^ mice were more susceptible to BSI than WT mice on day 3 post-IAV, but less susceptible on day 7 post-IAV (9). Because our results thus far indicate that IFNAR2 is important for anti-viral immunity to IAV, we next sought to determine whether the increased IAV-susceptibility of *Ifnar2*^−/−^ mice affected their BSI susceptibility, and whether *Ifnar2*^−/−^ mice exhibit a similar pattern of BSI susceptibility as *Ifnar1*^−/−^ mice. To this end we found that *Ifnar2*^−/−^ mice superinfected with *S. aureus* on day 3 post-IAV had comparable lung bacterial burden to *Ifnar2*^−/−^ mice infected with *S. aureus* alone (Figure 3A). Bacterial burden of either the BSI-*Ifnar2*^−/−^ mice or the *Ifnar2*^−/−^ mice infected with *S. aureus* alone did not significantly differ from that of *S. aureus*-only infected WT control mice. This suggested that IFNAR2 deficiency has no effect on host susceptibility to respiratory infection with *S. aureus*, whether introduced alone or as BSI on day 3 post-IAV. Because we previously found that IAV-infected *Ifnar1*^−/−^ mice were more susceptible to day 3 BSI than either their mock-inoculated littermates or IAV-infected WT mice (9), collectively our results indicate that presence of IFNAR1 is sufficient to protect mice from altered BSI susceptibility at day 3 post-IAV. Previously we reported that IFNAR1 deficiency had no effect on either lung viral burden or influenza disease progression both prior to and following BSI (9). In contrast to these findings in *Ifnar1*^−/−^ mice, here we found that lung virus burden of *Ifnar2*^−/−^ mice was significantly higher than that of WT mice both prior and 24 h after BSI induced on day 3 post-IAV (Figures 1C, 3B). This suggested that despite increased IAV susceptibility of mice in the absence of IFNAR2, BSI early after IAV infection did not exacerbate IAV disease in *Ifnar2*^−/−^ mice. Along these lines, BSI at day 3 post-IAV did not further exacerbate body weight loss and morbidity of IAV-infected *Ifnar2*^−/−^ mice (Figures 3C,F), further indicating that IFNAR2 may be more important for viral immunity than bacterial immunity. When compared to BSI-WT mice the BSI-*Ifnar2*^−/−^ mice had similar levels (Figure 3D) and types (Figure 3E) of cells recruited to the lung during BSI, indicating that the *Ifnar2*^−/−^ mice do not have a defect in cellular responses during a BSI. The superinfected *Ifnar2*^−/−^ mice also did not have significantly altered LDH (Figure 3G) or albumin (Figure 3H) levels in the BALF compared to WT mice. This suggests that at day 3 BSI with *S. aureus* the absence of IFNAR2 did not have a substantial effect on damage, similar to what we found early during IAV infection alone (Figure 1). Importantly, although IFNAR2 is not required for protection from day 3 BSI post-IAV, our results thus far demonstrate that when examined individually, the IFNAR subunits can lead to distinct outcomes.

### *Ifnar2^−/−^* mice have reduced susceptibility to day 7 BSI Post-IAV

**Figure 4:**
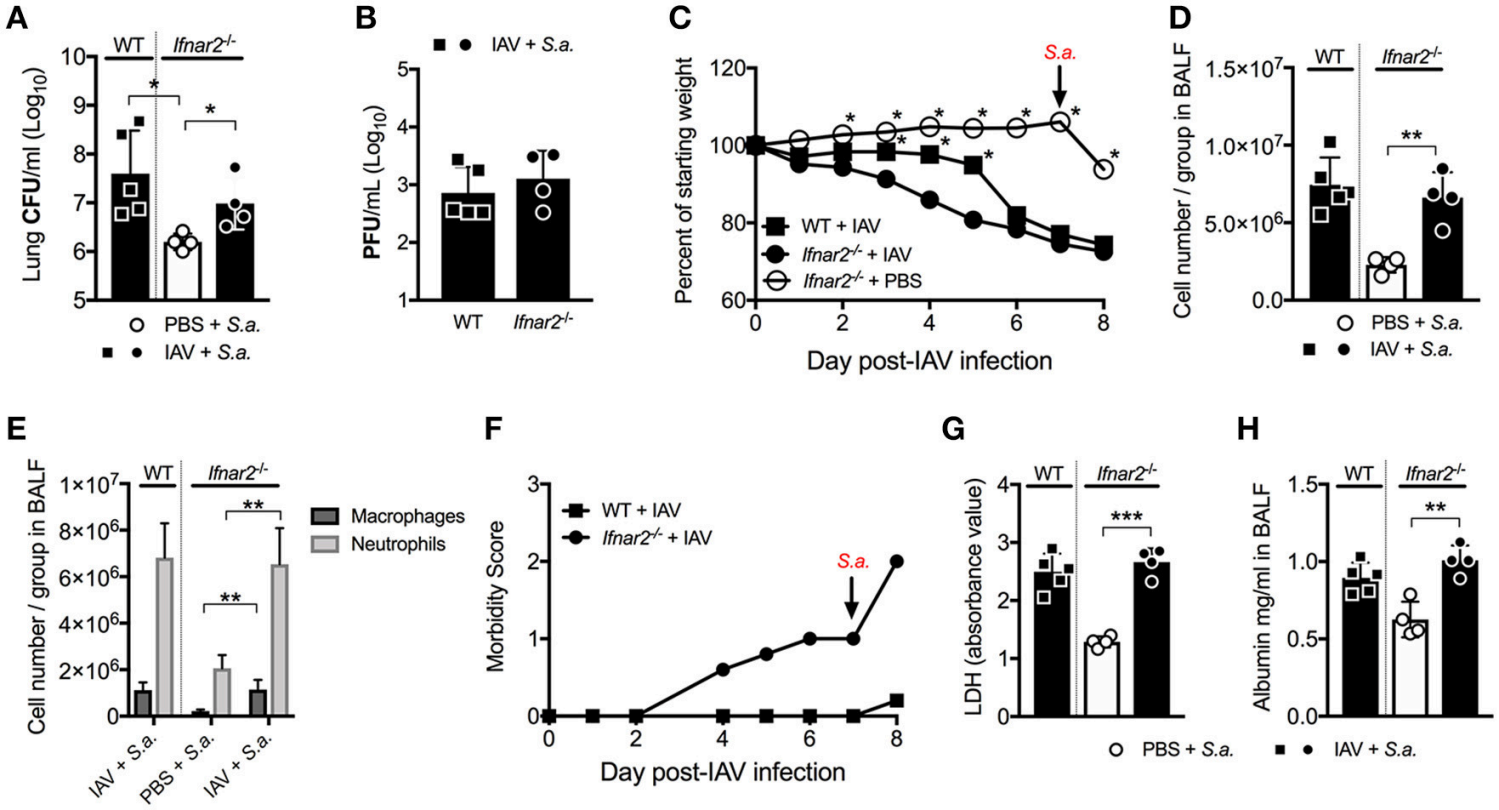
*Ifnar2*^−/−^ mice have reduced susceptibility to day 7 BSI post-IAV. WT and *Ifnar2*^−/−^ mice were infected with IAV or PBS on day 0 and challenged with *S. aureus* on day 7. Lung CFUs **(A)** and PFUs **(B)** were determined 24 h post-*S.a*.-challenge. **(C)** Weights were monitored daily and are represented as percent of starting body weight depicted as average/group (Significance represents WT+IAV or *Ifnar2*^−/−^ compared to *Ifnar2*^−/−^+IAV). Cell number **(D)** and differential counts **(E)** were determined from cell-free BALF 24 h post- *S.a*.-challenge. **(F)** Morbidity was assessed and monitored daily. Average daily score for each group is depicted. LDH **(G)** and Albumin **(H)** were analyzed in the cell-free BALF collected at 24 h post- *S.a*.-challenge. Data shown are mean ± SD results of ≥4 animals per group from one representative experiment. ***P >0.001; **P >0.01; *P > 0.05.

Unlike BSI at day 3 post-IAV, mice and humans are known to be more susceptible to BSI around day 7 post-IAV infection. Our laboratory and others have shown this susceptibility to depend on IFNAR1 with *Ifnar1*^−/−^ mice being less susceptible to BSI at day 7 post-IAV compared to WT mice (7, 9, 20, 21). Thus, we next sought to determine whether IFNAR2 plays a similar role as IFNAR1 in the susceptibility to day 7 post-IAV BSI. *Ifnar2*^−/−^ mice, similar to what we found for *Ifnar1*^−/−^ mice (9), were protected from BSI at day 7 post-IAV as compared to *S. aureus-Ifnar2*^−/−^ mice (Figure 4A). Like mice deficient in IFNAR1 signaling (9), when compared to WT mice *Ifnar2*^−/−^ mice had similar levels of virus following BSI at day 7 post-IAV (Figure 4B). While *Ifnar2*^−/−^ and WT mice had similar virus burden at 24 h post-BSI (Figure 4B), singly infected *Ifnar2*^−/−^ mice at day 7 post-IAV infection showed 1 log increase in the lung viral burden when compared to IAV-only infected WT mice (Figure 1C). This suggests that in the absence of IFNAR2, superinfection on day 7 prevents further increases in viral burden. Although the IAV infected *Ifnar2*^−/−^ mice had increased early weight loss compared to WT mice prior to BSI on day 7 (Figure 4C), these mice had a similar percentage of weight loss in response to *S. aureus* challenge (Figure 4C). However, IAV infected *Ifnar2*^−/−^ mice had increased morbidity compared to IAV infected WT mice both before and after challenge with *S. aureus* (Figure 4F), further suggesting that IFNAR2 signaling may be involved in regulating anti-viral immunity. At 24 h after day 7 BSI, WT, and *Ifnar2*^−/−^ mice had similar numbers (Figure 4D) and types (Figure 4E) of cells in the lung. This again was interesting because at day 7 post-IAV infection, *Ifnar2*^−/−^ mice showed an increase in number of neutrophils when compared to IAV-infected WT mice at that time (prior to *S. aureus* challenge; Figure 1D). This suggests that the increased number of neutrophils recruited during IAV and prior to BSI, may contribute to the decreased susceptibility of the *Ifnar2*^−/−^ mice to BSI at day 7, as neutrophils are known to be involved in anti-*S. aureus* immunity (22, 23). Similar to BSI at day 3 post-IAV, BSI at day 7 post-IAV did not significantly increase levels of LDH (Figure 4G) or albumin (Figure 4H) in the lung. These results indicate that like IFNAR1, presence of IFNAR2 is detrimental to BSI at day 7 post-IAV. These results also suggest that response to bacterial challenge may be beneficial for the late anti-viral response in the *Ifnar2*^−/−^ mice.

### *Ifnar2^−/−^* mice have decreased inflammatory cytokines at day 3 and increased IFNγ at day 7 Post-IAV BSI

**Figure 5:**
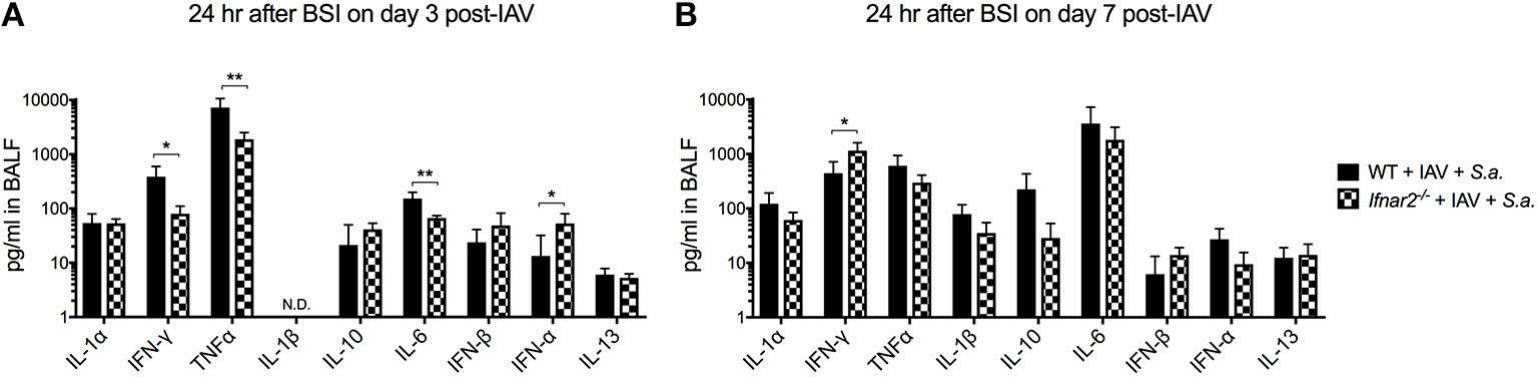
*Ifnar2*^−/−^ mice have decreased inflammatory cytokines at day 3 and increased IFNy at day 7 post-IAV BSI. WT and *Ifnar2*^−/−^ mice were infected with IAV or PBS on day 0 and challenged with *S. aureus* on day 3 **(A)** or day 7 **(B)**. Indicated cytokines were measured in cell-free BALF 24 h after *S.a*. challenge on day 3 post-IAV. N.D. is not detectable. **P >0.01; *P > 0.05.

Since IFNAR2 deficiency had no effect on numbers and types of recruited cells in response to BSI either at day 3 or day 7 post-IAV, we next sought to determine whether cytokines produced by WT and *Ifnar2*^−/−^ mice after BSI contribute to differences in BSI severity. In response to day 3 BSI, *Ifnar2*^−/−^ mice had less IFNγ, TNFα, and IL-6 compared to BSI-WT mice, indicating that the *Ifnar2*^−/−^ mice develop a less inflammatory lung environment (Figure 5A). The reduction in inflammatory cytokines in response to BSI at day 3 in *Ifnar2*^−/−^ mice did not correspond to less cells or less damage (Figures 3D,G), suggesting there is another mechanism involved. Moreover, the reduction in inflammatory cytokines we found in day 3 BSI-*Ifnar2*^−/−^ mice (Figure 5A) did not occur in *Ifnar2*^−/−^ mice in response to single infection with *S. aureus* alone (Supplemental Figure 1). As it relates to interferon responses, the *Ifnar2*^−/−^ mice also had increased IFN-α on day 3 BSI compared to WT mice. In response to day 7 BSI, *Ifnar2*^−/−^ mice only had an increase in IFNγ compared to BSI-WT mice (Figure 5B). These results suggest that the decreased susceptibility of *Ifnar2*^−/−^ mice to day 7 BSI is not due to an altered lung environment as there were no major changes in cytokine levels associated with anti-viral or anti-bacterial immunity. Together, these results suggest that the inflammatory environment on day 3 and day 7 may be more related to IAV outcome than the outcome of BSI or single *S. aureus* infection.

### STAT3 contributes to the increased susceptibility of *Ifnar1^−/−^* mice, but not *Ifnar2^−/−^* mice to day 3 BSI Post- IAV

**Figure 6:**
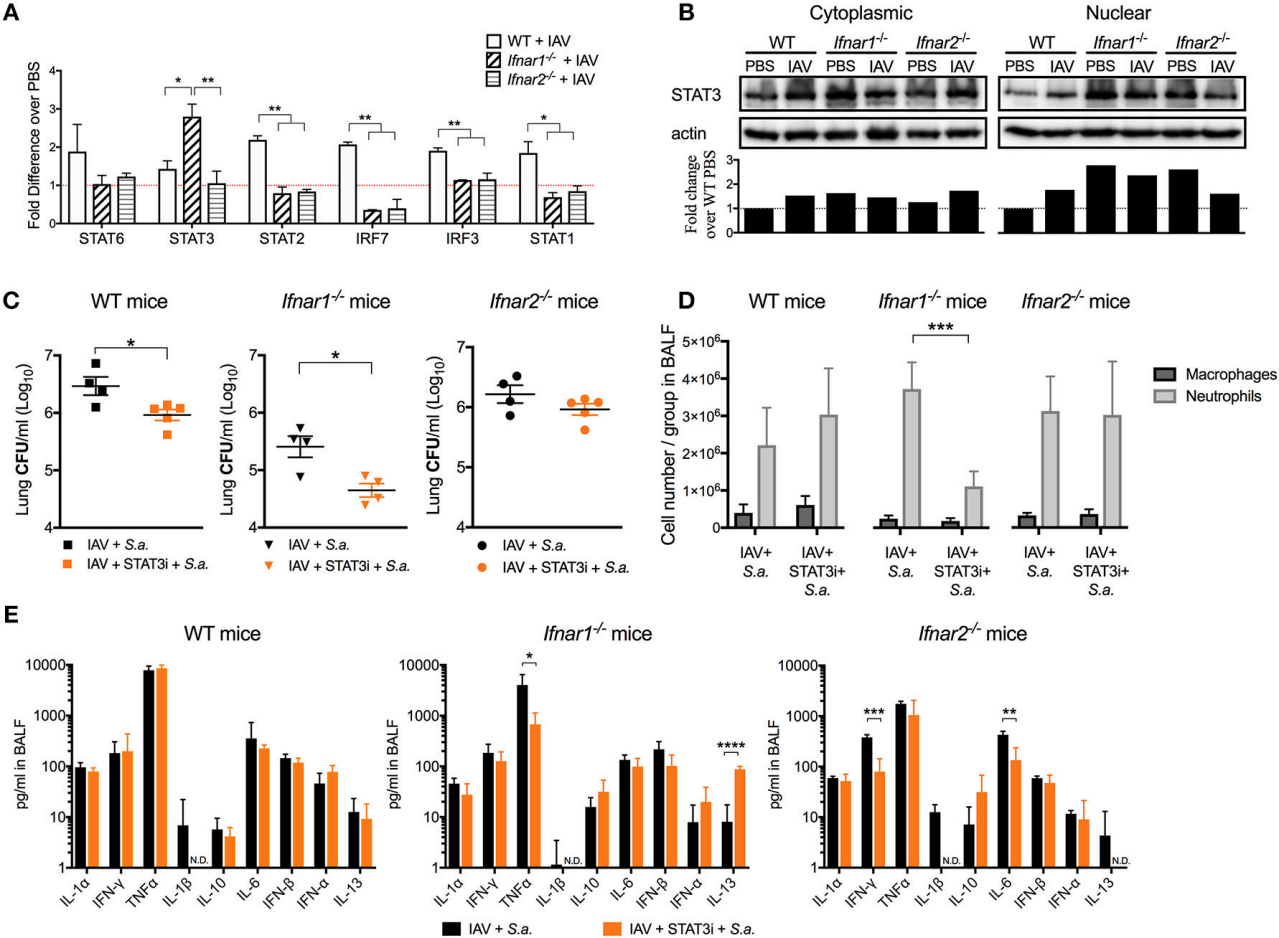
STAT3 contributes to the increased susceptibility of day 3 BSI-*Ifnar1*^−/−^ mice post-IAV. **(A)** WT, *Ifnar1*^−/−^, and *Ifnar2*^−/−^ mice were infected with IAV or PBS on day 0 and gene expression from RNA isolated from the whole lung was analyzed. **(B)** STAT3 protein was analyzed from cytoplasmic and nuclear fractions isolated from WT, *Ifnar1*^−/−^, and *Ifnar2*^−/−^ primary alveolar epithelial cells infected with IAV or inoculated PBS for 24 h. STAT3 was normalized to b-actin protein level and fold change over WT-PBS was calculated (Whole western blots: Supplemental Figures 2–5). **(C–E)** WT, *Ifnar1*^−/−^, and *Ifnar2*^−/−^ mice were infected with IAV on day 0, inoculated with STAT3 inhibitor or PBS 6 h post-IAV, and challenged with *S. aureus* on day 3. **(C)** Lung CFUs, **(D)** differential counts from the cell-free BALF, and **(E)** indicated cytokines from cell-free BALF were analyzed 24 h post- *S.a*.-challenge. Data shown are mean ± SD results of ≥4 animals per group from one representative experiment. N.D. is not detectable. ***P >0.001; **P >0.01; *P > 0.05.

IFN-β and the IFN-α's are known to have different affinities for IFNAR1 and IFNAR2 and to induce different gene expression profiles depending on their concentration and timing (11, 24). Thus, we next wanted to determine whether engagement of either IFNAR subunit by IAV resulted in induction of distinct anti-viral pathways. We found that both IFNAR subunits were required for the early expression of STAT1/2 and IRF3/7 to the levels found in WT mice (IFNAR^+/+^) following viral recognition (Figure 6A). Interestingly, early IAV infection in *Ifnar1*^−/−^ mice resulted in a significant increase in STAT3 expression in the lung compared to both WT and *Ifnar2*^−/−^ mice. This increase in expression of STAT3 in *Ifnar1*^−/−^ mice corresponded to the high level of nuclear STAT3 protein found in *Ifnar1*^−/−^ primary pulmonary epithelial cells regardless of IAV infection (Figure 6B). This indicated that STAT3 activation in *Ifnar1*^−/−^ mice may have a role in early anti-viral immunity and subsequent BSI severity at day 3 in these mice. To address this possibility, we treated the IFNAR subunit-knockout mice with a STAT3 phosphorylation inhibitor (15) 6 h after IAV infection and determined their susceptibility to BSI at day 3 post-IAV. We found that P-STAT3 inhibition in both the WT and *Ifnar1*^−/−^ mice resulted in reduced susceptibility to BSI, 0.5 log and 1 log (Figure 6C), respectively, compared to untreated littermate mice. Additionally, we found that P-STAT3 inhibition did not alter susceptibility of *Ifnar2*^−/−^ mice to BSI on day 3, indicating that IFNAR1 and IFNAR2 induce disparate mechanisms in response to viral infection. Upon analyzing the cell abundances in the BALF of the WT, *Ifnar1*^−/−^ and *Ifnar2*^−/−^ mice we only found the P-STAT3-inhibited *Ifnar1*^−/−^ mice to have decreased levels of neutrophils (Figure 6D). To determine whether P-STAT3 inhibition altered the anti-viral state of mice at 24 h after day 3 BSI, we analyzed their cytokine profiles. When compared to untreated littermates, the P-STAT3 inhibition did not alter cytokines produced by WT mice (Figure 6E), but resulted in decreased IFNγ and IL-6 in *Ifnar2*^−/−^ mice (Figure 6E). The decreased IFNγ and IL-6 in P-STAT3-inhibited *Ifnar2*^−/−^ mice did not appear to significantly affect BSI outcome in these mice (Figure 6C). In *Ifnar1*^−/−^ mice superinfected on day 3, P-STAT3 inhibition resulted in decreased TNFα and a 10-fold increase in IL-13 levels compared to untreated superinfected *Ifnar1*^−/−^ mice. These results are consistent with our previous findings that treatment with mrIL-13 reversed the increased day 3 BSI susceptibility and resulted in decreased neutrophil accumulation in *Ifnar1*^−/−^ mice (9). Therefore, our results expand our previous findings by demonstrating that STAT3 contributes to increased susceptibility of *Ifnar1*^−/−^ mice to BSI at day 3 post-IAV at least in part by inhibition of IL-13 production.

### IFN-β signaling through *Ifnar2* rescued mice from morbidity and mortality upon lethal IAV infection

**Figure 7:**
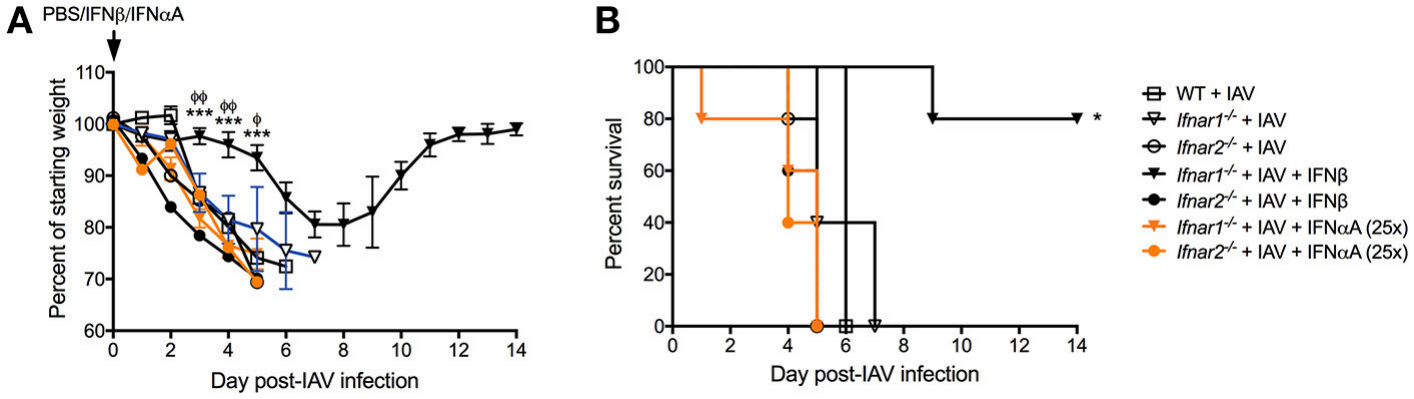
IFN-β can rescue influenza-mediated morbidity and mortality in *Ifnar1*^−/−^ mice. WT, *Ifnar1*^−/−^, and *Ifnar2*^−/−^ mice were infected with IAV or PBS on day 0 and treated with PBS, rIFN-β, or rIFN-αA at 0, 3, and 6 h post-IAV. **(A)** Weights were monitored daily and are represented as percent of starting body weight depicted as average/group (Significance represents *Ifnar1*^−/−^+IAV+β compared to *WT or ^φ^*Ifnar1*^−/−^+IAV). **(B)** Survival is represented as percent survival/group Data shown are mean ± SD results of ≥4 animals per group from one representative experiment. ***P > 0.001; ^φφ^*P* > 0.01, ^φ^*P* > 0.05.

Our results thus far indicate that engagement of either IFNAR subunit individually differentially shapes both anti-IAV immunity and BSI susceptibility. These two IFNAR subunits are known to bind IFN-α's and IFN-β with different affinity inducing distinct gene profiles (11, 25). We found that IFN-αA, but not IFN-β inhibited production of IL-13 resulting in increased host susceptibility to BSI and that IFN-αA was involved in mediating increased susceptibility to *S. aureus* (9). Therefore, here we sought to determine whether IFN-αA and IFN-β have different effects on protection from IAV infection while signaling via distinct IFNAR subunits. IFNαA treatment of mice deficient in either IFNAR subunit (Figure 7A orange traces) had no effect on IAV-induced body weight loss when compared to either their untreated littermates or WT mice (open symbols). Treatment of mice that have functional IFNAR2 (*Ifnar1*^−/−^ mice) with mrIFN-β (black triangles) protected these mice from morbidity and increased their survival compared to IAV-infected littermates (open triangle) or IAV-infected WT mice (open squares) (Figures 7A,B). Treatment of mice without functional IFNAR2 (*Ifnar2*^−/−^ mice) with mrIFN-β (black circles) did not protect these mice from morbidity or mortality when compared to mrIFN-treated *Ifnar1*^−/−^ mice. The mrIFN-β treatment of *Ifnar2*^−/−^ mice however, did slightly but not significantly accelerate and worsen body weight loss when compared to untreated *Ifnar2*^−/−^ mice (open circles). These results indicate that presence of IFNAR2 and signaling by IFN-β are sufficient to induce a protective anti-IAV state. Our results also suggest that IFN-β produced in the *Ifnar1*^−/−^ and WT mice following IAV infection (Figures 2A, 6A) is not sufficient to provide protection and that the protective effects only occur when IFN-β is present at the very beginning of viral infection (9).

## Discussion

Type I IFNs play a role in determining influenza and BSI severity. While cell surface receptor for type I IFNs consists of two distinct subunits (IFNAR1 and IFNAR2), almost all research on determining the role of type I IFNs in susceptibility to influenza and subsequent BSIs has focused solely on the involvement of IFNAR1 (using *Ifnar1*^−/−^ mice). To this end our lab and others have shown that IFNAR1 is important in regulating time-dependent susceptibility to BSI during IAV infection (4, 7, 9). In the last decade, a number of reports demonstrated the ability of IFNAR1 and IFNAR2 to bind individual IFNs independent of one another, with different affinities, and subsequently causing induction of different gene (11, 24, 25). These reports alluded that the receptor subunits have distinct functions in relation to disease outcomes (25). Here, we begin to unravel how the absence of IFNAR2 affects IAV and BSI severity.

Our study revealed that IFNAR2 is required for effective anti-IAV immune responses, particularly as it relates to protection from influenza-mediated morbidity and mortality. While our results combined with work by others (5) suggest that a complete IFNAR receptor is important for protection from influenza, the accelerated morbidity and mortality, as well as increased viral burden of the *Ifnar2*^−/−^ mice indicate that IFNAR2 plays a larger role than IFNAR1 in regulating the anti-viral response. That IFNAR2 deficiency resulted in increased neutrophil recruitment and an increased level of LDH production by day 7 of IAV infection further suggests that IFNAR2 may also control damage response during viral infections. Further studies are necessary to elucidate the exact mechanism by which IFNAR2 regulates anti-IAV immunity.

As it relates to host susceptibility to post-IAV BSIs, we demonstrate that IFNAR2 has both similar and distinct roles in BSI susceptibility compared to IFNAR1. Specifically, we found that IFNAR1 signaling in the absence of IFNAR2 (*Ifnar2*^−/−^ mice) was sufficient to prevent the increased BSI susceptibility that we previously found to occur in *Ifnar1*^−/−^ mice at day 3 post-IAV (9), suggesting that IFNAR1 alone is able to control bacterial burden more than IFNAR2 alone at day 3 post-IAV. In regards to BSI induced at day 7 post-IAV, *Ifnar2*^−/−^ mice shared the same decrease in susceptibility as has been previously found for *Ifnar1*^−/−^ mice (4, 9). As WT mice are more susceptible to day 7 BSI than either IFNAR subunit knockout mice, our combined results from *Ifnar2*^−/−^ mice and previous results from *Ifnar1*^−/−^ mice (4, 7, 9) suggest that a complete IFNAR is required for the increased susceptibility phenotype and that absence of either receptor subunit is sufficient to provide protection.

Conventional type I IFN signaling utilizes STAT1/2 heterodimer (26). A side by side comparison of intracellular signaling molecules known to be engaged in type I IFN cascade revealed preferential engagement of STAT3 upon IAV infection of *Ifnar1*^−/−^, but not *Ifnar2*^−/−^ or WT mice. We found that this engagement of STAT3 contributed to the increased BSI susceptibility of *Ifnar1*^−/−^ mice at day 3 post-IAV. Our previous work demonstrated that the *Ifnar1*^−/−^ day 3 BSI phenotype was at least in part due to the absence of IL-13 as increased susceptibility to BSI at that time post-IAV could be reversed by treating the *Ifnar1*^−/−^ mice with mrIL-13, leading to a reduction in neutrophils and bacterial burden (9). Here, we found that P-STAT3 inhibition in *Ifnar1*^−/−^ mice prior to day 3 BSI similarly reduced the level of neutrophil recruitment and caused a 10-fold increase in IL-13 in response to day 3 BSI. STAT3 has been previously reported to be involved in regulating inflammatory mediators and subsequent neutrophil trafficking during infection, where inhibition of STAT3 reduced neutrophil chemokines and recruitment (27, 28). During viral infection, type I IFN signaling through IFNAR induces STAT1/STAT2 activity, but also leads to the induction of STAT3, which is thought to provide negative feedback keeping the IFN response under control (29). STAT3 deficiency was found to enhance anti-viral activity and gene expression in response to type I IFNs (30). Thus, it is tempting to speculate that in the presence of IFNAR2, the STAT3 activation in IAV-infected *Ifnar1*^−/−^ mice does not allow for induction of an anti-IAV immune response. Our data imply that STAT3 induction in *Ifnar1*^−/−^ mice may be involved in preventing the increase in IL-13 that is required for controlling neutrophil recruitment and bacterial killing. How IFNAR1- or IFNAR2-induced STAT3, whether in conjunction with STAT1 or other STATs, is involved in viral and BSI susceptibility remains unknown.

Differential signaling through a single cytokine receptor is not a new concept in immunology. Results of interactions between cytokines and their cognate receptors can vary depending on the specific tissue and cellular environment, availability of the substrate and/or receptor, and affinity and avidity of the interactions (31). There are multiple examples where differential cellular processes induced by different cytokines occurs through a shared common receptor (31–33). As for type I IFNs, IFN-β, and the IFN-α's have been shown to induce distinct signaling pathways depending on their abundance, with all IFNs inducing “robust” genes (anti-viral) at low concentrations, and only IFN-β inducing “tunable” (anti-proliferative) at higher, still physiologic, concentrations (34–36). Importantly, de Weerd and colleagues established that type I IFNs are able to bind the individual IFNAR subunits independently (11). Specifically, they found that IFN-β ligates IFNAR1 independently of IFNAR2 and also the opposite, that IFN-β can ligate IFNAR2 independently of IFNAR1. However, human IFNα2, which shares high homology with mouse IFN-αA, was only able to form a stable complex with the extracellular domain of IFNAR2, which is the high-affinity portion of the IFNAR receptor. Here, we demonstrate that the morbidity and mortality associated with IAV infection of mice lacking IFNAR1 can be rescued by the administration of mrIFN-β at the time of IAV infection, but IFN-β treatment did not rescue mice lacking IFNAR2. These results suggest that the presence of IFNAR2 is required to generate a protective anti-viral response to IAV infection when stimulated with IFN-β, but that presence of IFNAR1 is not. Which response, whether it be the robust or tunable, and which of those genes are important for providing protection to IAV infection by IFNAR2 in the *Ifnar1*^−/−^ mice will provide insight into how these receptor subunits are regulating the immune response to IAV. A better understanding of how the IFN-β-IFNAR2 complex in the absence of IFNAR1 can lead to protection will improve our knowledge of viral immunity.

Collectively our results demonstrate that either IFNAR subunit is sufficient for interferon signaling *in vivo*. As we began to elucidate differences in how the individual subunits shape the anti-viral immune response we found that IFNAR2 plays a non-redundant role in induction to anti-viral immunity. As such, we found IFNAR2 to be essential for both decreasing the morbidity and mortality associated with IAV infections and in altering subsequent host susceptibility to BSI. While further studies will determine the intracellular signaling mechanisms utilized by individual IFNAR subunits and whether these subunits have distinct outcomes in other viral and bacterial infections, results presented here set a stage for these mechanistic studies by emphasizing the importance of understanding the contributions of the entire receptor to disease outcomes.

## Ethics statement

All animal experiments were approved by the Montana State University Institutional Animal Care and Use Committee.

## Author contributions

KMS designed and performed experiments, analyzed data, and contributed to writing of the paper. KL and LJ designed and performed experiments and analyzed data. KS, HC, JW, and HH performed experiments. AR-A designed experiments, analyzed data, and contributed to writing of the paper.

### Conflict of interest statement

The authors declare that the research was conducted in the absence of any commercial or financial relationships that could be construed as a potential conflict of interest.
