# Supplementary material for: IFNAR2 Is Required for Anti-influenza Immunity and Alters Susceptibility to Post-influenza Bacterial Superinfections
Source: Front Immunol. 2018 Nov 9;9:2589. doi: 10.3389/fimmu.2018.02589 (PMC6237881; doi:10.3389/fimmu.2018.02589)
Supplement: Supplementary file 1 [file Data_Sheet_1.PDF]

Supplemental Figures 1-4

STAT3 and beta-actin proteins were analyzed from cytoplasmic and nuclear fractions isolated from WT (lanes 1-3), Ifnar1-/- (lanes 4-6), and Ifnar2-/- (lanes 7-9) primary alveolar epithelial cells infected with IAV (lanes 3, 6, 9) or inoculated PBS (lanes 1, 4, 7) for 24 hr. (Lanes 2, 5, 8 are samples not related to this manuscript and were not included)

Supplemental Figure 1 – STAT3 cytoplasmic

Supplemental Figure 2 – beta-actin cytoplasmic

Supplemental Figure 3 – STAT3 nuclear

Supplemental Figure 4 – beta-actin nuclear
