# Supplementary figures and images for: IFNAR2 Is Required for Anti-influenza Immunity and Alters Susceptibility to Post-influenza Bacterial Superinfections
Source: Front Immunol. 2018 Nov 9;9:2589. doi: 10.3389/fimmu.2018.02589 (PMC6237881; doi:10.3389/fimmu.2018.02589)

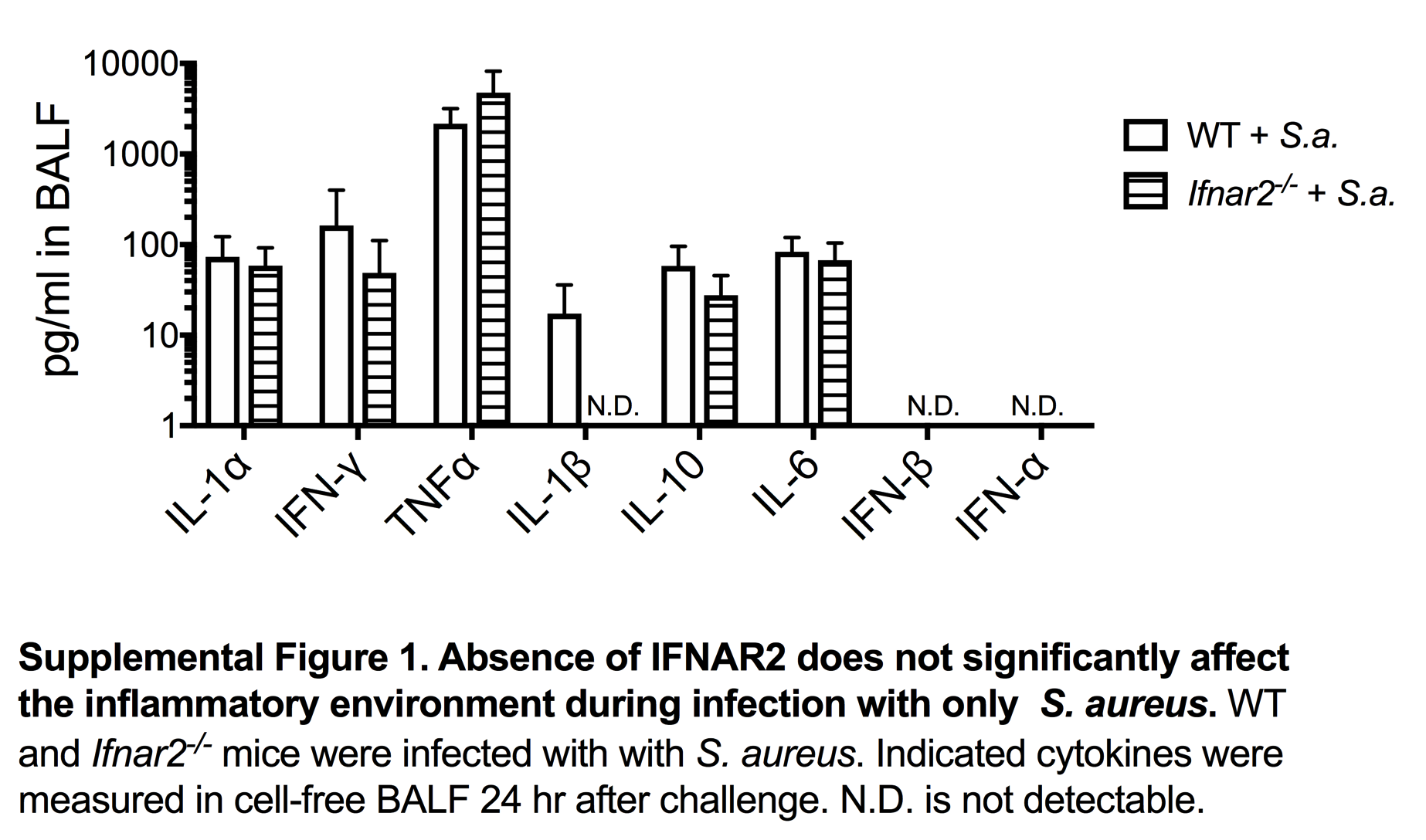

Supplement: Supplementary file 2 [file Image_1.tiff]

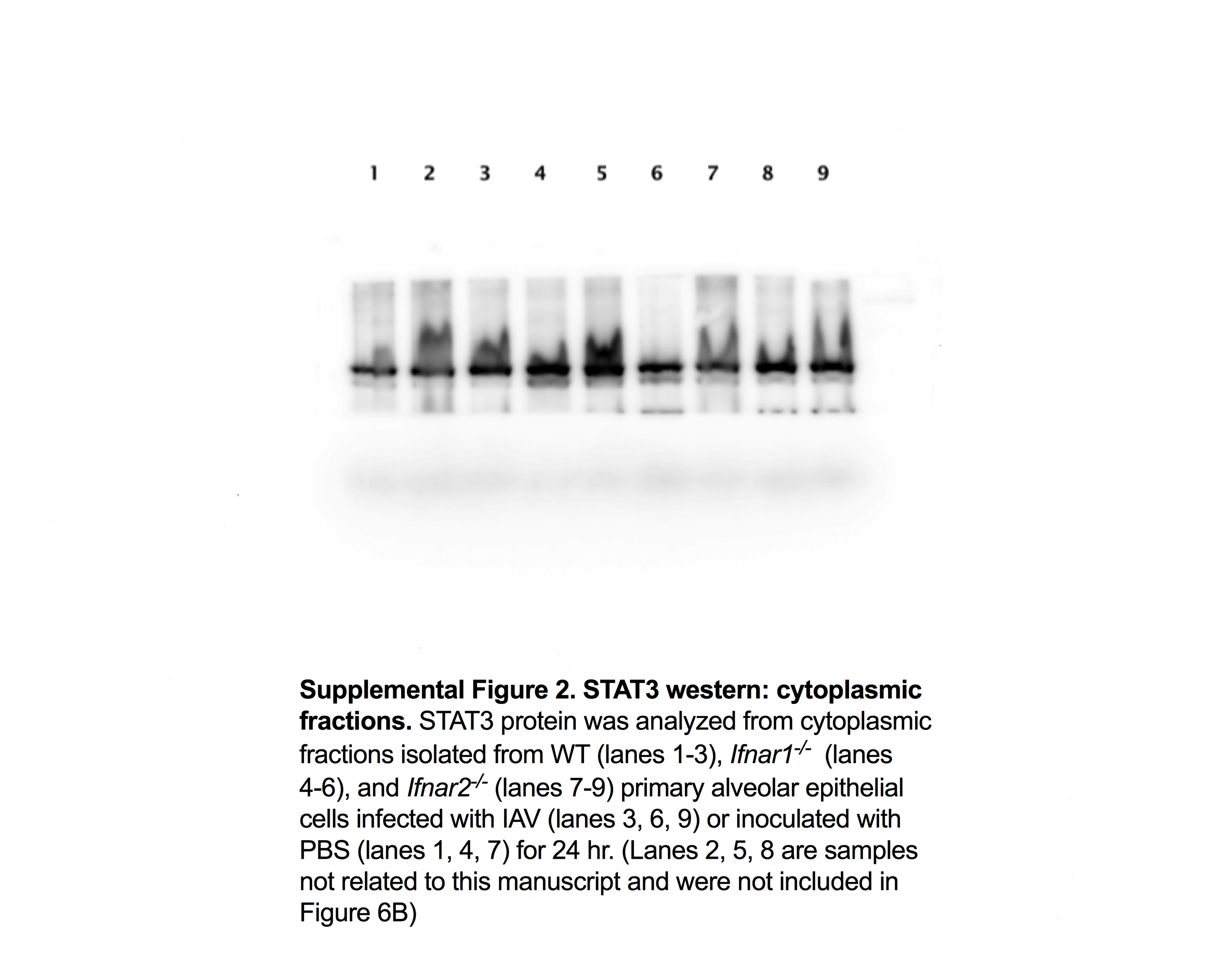

Supplement: Supplementary file 3 [file Image_2.tiff]

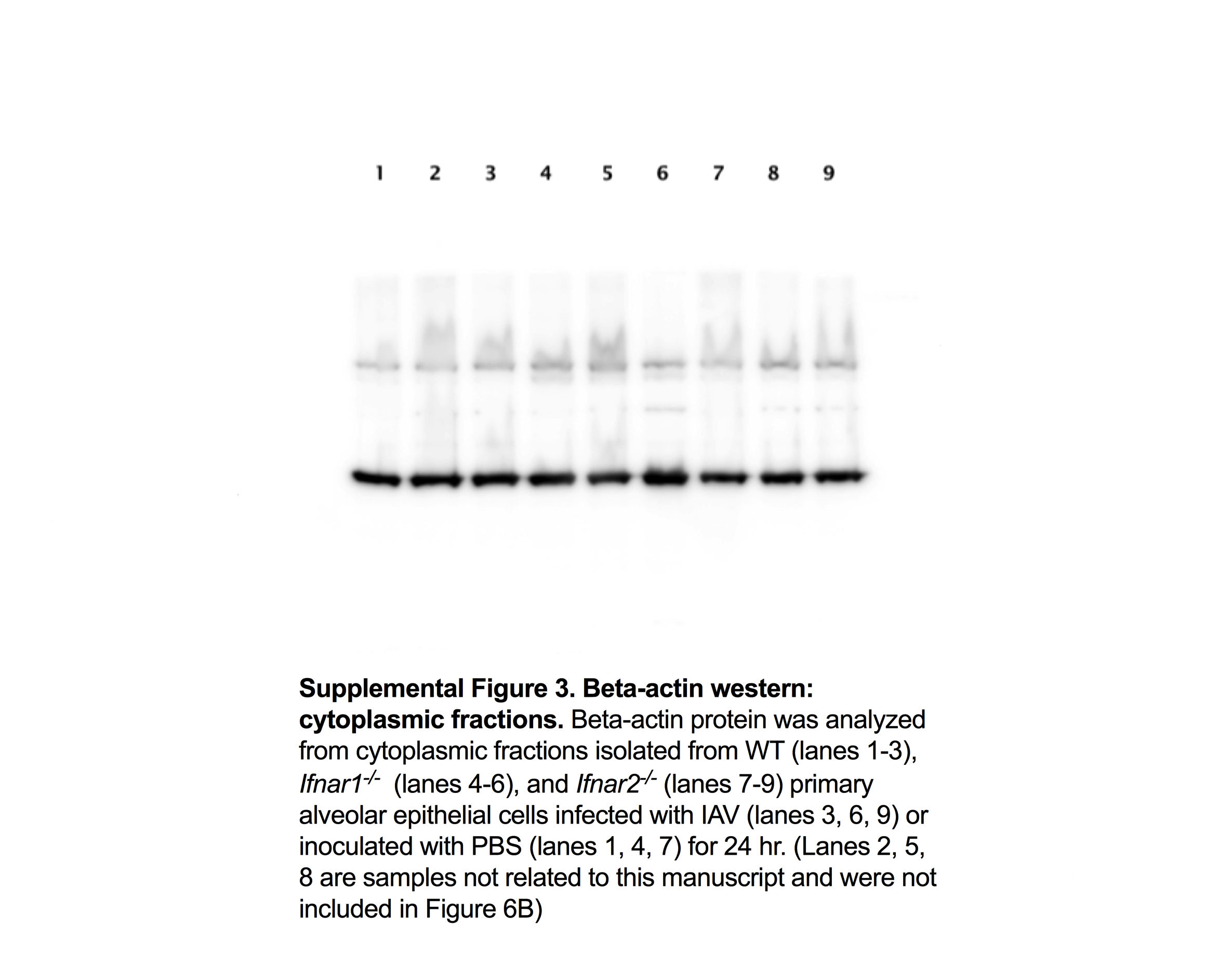

Supplement: Supplementary file 4 [file Image_3.tiff]

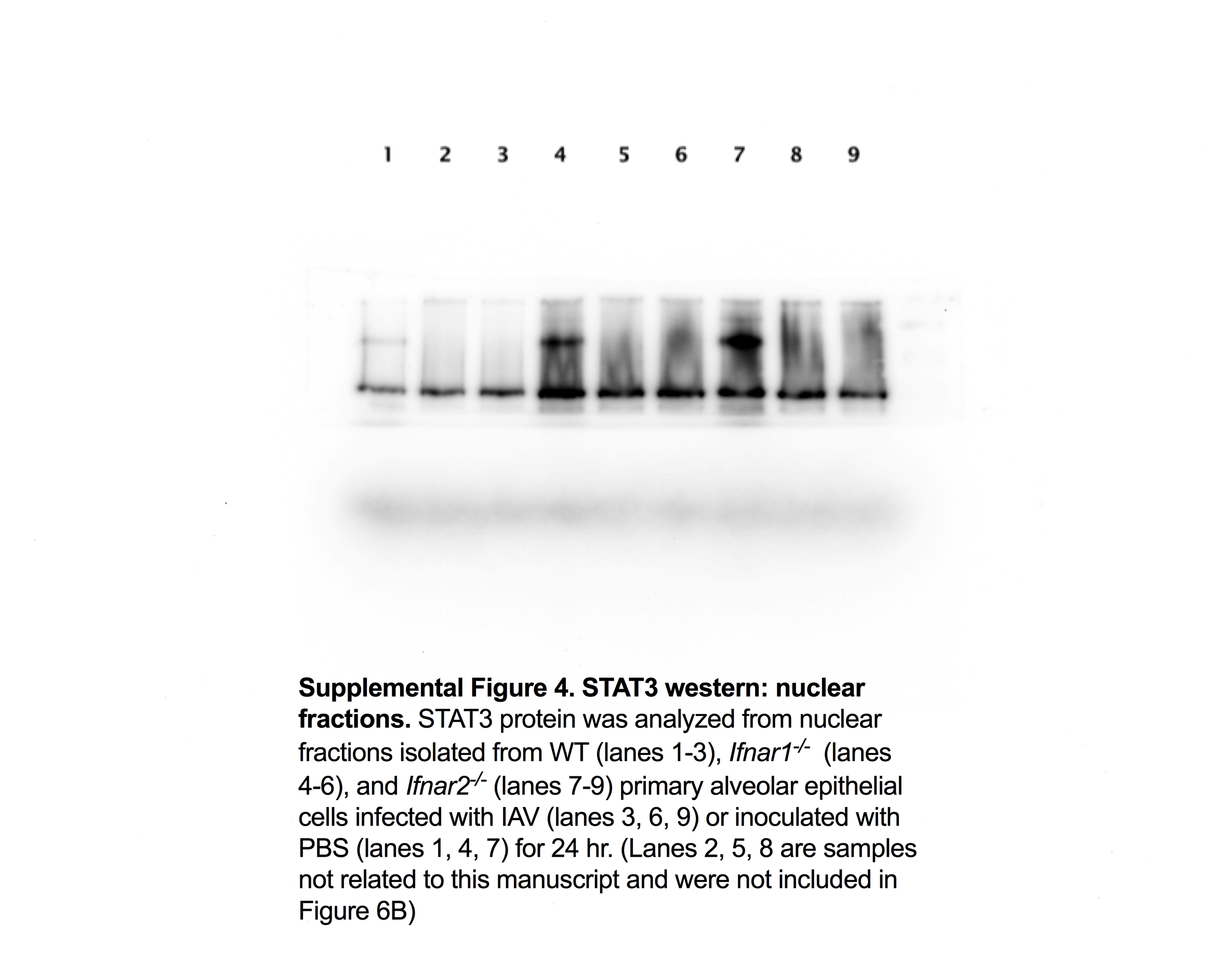

Supplement: Supplementary file 5 [file Image_4.tiff]

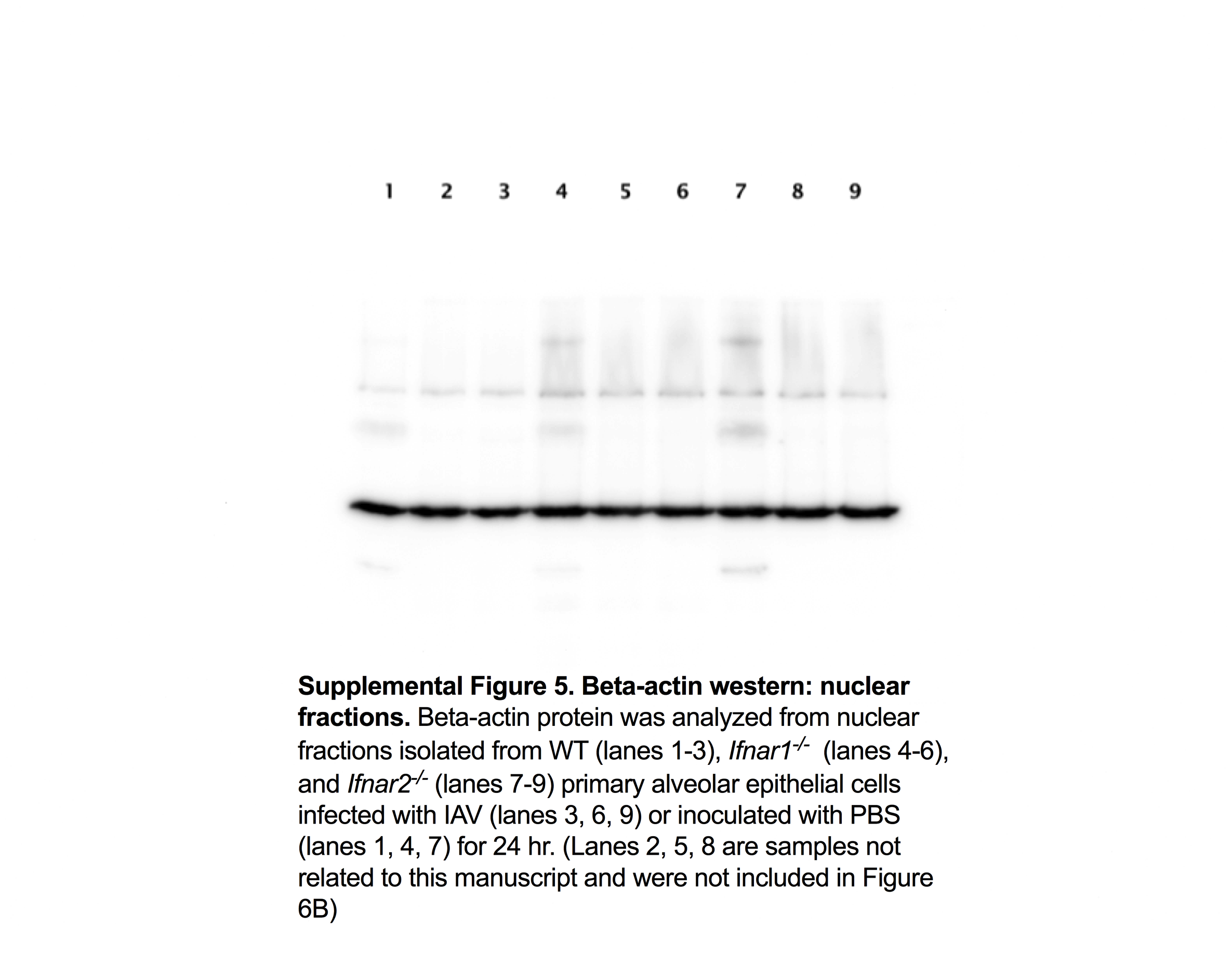

Supplement: Supplementary file 6 [file Image_5.tiff]
